# A Spatial Crowdsourcing Engine for Harmonizing Volunteers’ Needs and Tasks’ Completion Goals

**DOI:** 10.3390/s24248117

**Published:** 2024-12-19

**Authors:** Maite Puerta-Beldarrain, Oihane Gómez-Carmona, Liming Chen, Diego López-de-Ipiña, Diego Casado-Mansilla, Felipe Vergara-Borge

**Affiliations:** 1 Deustotech, University of Deusto, Avda. Universidades 24, 48007 Bilbao, Spain; oihane.gomezc@deusto.es (O.G.-C.); felipe.vergara@deusto.es (F.V.-B.); 2 School of Computer Science and Technology, Dalian University of Technology, Dalian 116024, China; limingchen0922@dlut.edu.cn; 3 Faculty of Engineering, University of Deusto, Avda. Universidades 24, 48007 Bilbao, Spain; dipina@deusto.es (D.L.-d.-I.); dcasado@deusto.es (D.C.-M.)

**Keywords:** citizen science, spatial crowdsourcing, crowdsensing, task allocation

## Abstract

This work addresses the task allocation problem in spatial crowdsensing with altruistic participation, tackling challenges like declining engagement and user fatigue from task overload. Unlike typical models relying on financial incentives, this context requires alternative strategies to sustain participation. This paper presents a new solution, the Volunteer Task Allocation Engine (VTAE), to address these challenges. This solution is not based on economic incentives, and it has two primary goals. The first one is to improve user experience by limiting the workload and creating a user-centric task allocation solution. The second goal is to create an equal distribution of tasks over the spatial locations to make the solution robust against the possible decrease in participation. Two approaches are used to test the performance of this solution against different conditions: computer simulations and a real-world experiment with real users, which include a qualitative evaluation. The simulations tested system performance in controlled environments, while the real-world experiment assessed the effectiveness and usability of the VTAE with real users. This research highlights the importance of user-centered design in citizen science applications with altruistic participation. The findings demonstrate that the VTAE algorithm ensures equitable task distribution across geographical areas while actively involving users in the decision-making process.

## 1. Introduction

Introduced by Howe in 2006 [1,2], crowdsourcing is a technique that involves non-professionals in the data-gathering stage of the scientific process [3]. Participants usually respond to open calls online, performing tasks that are more suited to humans than machines. One example is Amazon Mechanical Turk (https://www.mturk.com/ accessed on 15 December 2024), through which users perform tasks such as image identification, text transcription, surveys, or data classification. By leveraging collective intelligence, contributions, and collaboration, this solution can address complex issues that are challenging for conventional methods.

Advances in mobile devices have led to a new form of crowdsourcing: spatial crowdsourcing (SC). The primary distinction from conventional crowdsensing techniques lies in the spatial–temporal dependence of the tasks. Workers must be physically spread through diverse locations at different time periods for task completion [4]. Spatial crowdsourcing experiments involve gathering and utilizing a wide range of spatiotemporal data [5,6], offering a huge potential across a wide range of applications. These applications span various domains, including environmental data collection [7], transportation exemplified by Uber (https://www.uber.com/, accessed on 15 December 2024) and Didi (https://web.didiglobal.com/, accessed on 15 December 2024), journalism [8,9], and business intelligence platforms such as Gigwalk (https://www.gigwalk.com/, accessed on 15 December 2024) and TaskRabbit (https://www.taskrabbit.com/, accessed on 15 December 2024). For example, Uber operates as an unlicensed taxi-calling and peer-to-peer ride-sharing application through which users can either hire a taxi or share a ride. During its operation, the server matches the travel requests of users with service providers (drivers) based on their spatiotemporal context. The primary optimization goal is to enhance the service delivery rate and increase the earnings of the service providers. It considers constraints such as the driver’s maximum number of trip requests per day, the passenger’s pickup time, and vehicle preferences. Additional constraints, like the driver’s destination, are also considered to ensure that the pickup locations of trip requests align closely with the driver’s intended route. Each SC problem involves its own constraints and goals.

In the literature, solving spatial crowdsourcing problems typically involves a task assignment, through which tasks are allocated to users for execution [4]. Task allocation approaches are categorized into two types: platform-centric and user-centric. In platform-centric task allocation, the platform (or server) assigns tasks directly to users. Existing task allocation models predominantly focus on platform-centric approaches [10,11,12,13,14,15]. They are primarily concerned with maximizing profit for the platform by minimizing user rewards. On the other hand, in user-centric task allocation, users choose from a list of available tasks, and the primary objective remains profit-driven, with an emphasis on increasing user earnings [16,17,18,19,20,21,22,23,24,25,26]. Anyhow, all task allocation solutions consider the financial reward of the user as the primary strategy to motivate and promote participation.

Spatial crowdsourcing overlaps with citizen science (CS), which engages non-professionals in various stages of the scientific process. CS initiatives rely on the voluntary participation of proactive citizens driven by non-financial motivations, such as self-improvement, personal enjoyment, or altruism. CS problems require tailored approaches considering users’ preferences, behaviors, and willingness to invest effort, which cannot be assumed under economic-focused models [27].

The typical solution of the SC diverges from citizen science principles, which rely on altruistic participation. In addition, many applications, such as traffic, noise, and weather monitoring, typically do not offer financial rewards to the users involved [28]. Consequently, there is significant potential for developing task allocation strategies that prioritize enhancing user experience, thereby fostering more sustained and effective participation that is not based on financial rewards. Altruistic, unpaid participation poses new challenges, including the risk of sudden drops in participation, which may undermine data integrity and validity. Another challenge is the possible overloading of users, which could reduce their engagement [29]. Requesting continuous participation without immediate rewards could adversely affect user motivation and their sense of agency. Hence, task allocation resolutions in altruistic participation must be robust against decreased participation and avoid the over-exploitation of users.

Considering this, this work presents a task allocation solution named the Volunteer Task Allocation Engine (VTAE) for altruist participation, removing the financial rewards assumption of the literature. This solution must assign users to tasks independently of the spatial crowdsourcing problem and its requirements, meaning it must be agnostic to the SC problem. VTAE has two goals: (1) to improve user experience and (2) to create an equitable distribution of tasks over the static geographical locations of the SC problem. Related to the first goal, the task allocation algorithm adopts a user-oriented approach, considering the user’s location. This ensures users feel more integrated and valued in the task assignment process. In addition, the experiment design incorporates a workload limitation to regulate user effort and prevent overloading. Regarding the second objective, ensuring an even distribution of tasks across locations would safeguard the algorithm against sudden drops in participation, thereby preserving data integrity. To achieve this objective, the system handles incomplete tasks by reallocating them to other users, thereby preventing delays caused by unfulfilled user commitments.

This paper is organized as follows: Section 2 reviews existing research on task assignment solutions. Section 3 outlines the formulation of the spatial crowdsourcing (SC) problem addressed in this work. Section 4 details the proposed human-centric solution. Section 5 describes the experiments and metrics used to evaluate the performance of the Volunteer Task Allocation Engine (VTAE). Section 6 outlines the simulation experiments conducted to evaluate the performance of VTAE in a controlled environment. Section 7 presents the second evaluation, which is based on a real-world test to assess the adoption and adaptation of VTAE by users. The conclusion of these experiments is explained in Section 8. Finally, Section 9 discusses the need for a revised problem statement and summarizes the key findings of this study.

## 2. Related Work

Spatial crowdsourcing (SC) involves outsourcing location-dependent tasks to a large group of mobile workers who perform spatial tasks that involve traveling to specified locations [5], e.g., data-gathering or taking snapshots of an area. Spatial crowdsourcing employs task allocation to distribute tasks to appropriate workers in order to achieve specific goals while adhering to spatial, temporal, and other constraints.

A few studies have handled platform-centric task allocation. First, Li et al. proposed the ContinuousSensing framework, which is a human-robot task allocation model that facilitates cooperation and coordination between different types of robots and humans [10]. It also features a task migration model that transfers tasks from failed participants to other available participants. This approach improves the quality of crowdsensing task completion while maintaining relatively low task execution costs. Gao et al. developed a personalized task allocation strategy for mobile crowdsensing that minimizes costs by considering both user travel distance and task preferences [11]. It treats task allocation as a dynamic optimization problem, modeled as a complex version of the traveling salesman problem (TSP). Huang et al. proposed Optimized Allocation of Time-Dependent Tasks (OPAT), a time-dependent task allocation contribution that considers sensing duration and the user’s sensing capacity [12]. The goal of this approach is to obtain the most optimal solution, maximizing the sensing capacity of each mobile user. Zhan et al. investigated incentive mechanisms to motivate participation in platform-centric task allocation [14]. They employed a one-to-many bargaining framework that considers both the resource limitations and the resource needs of users.

On the other hand, other works have created user-centric task allocation. Yucel et al. created task allocation to satisfy system stability and user satisfaction [16]. They used task matching to minimize unhappy participant pairs while maintaining system stability. Cai et al. proposed user-centric algorithms based on valuation and distance from/to the task [17]. They calculate the payment according to the task performed. The task owner then selects the participants with the minimum payment. Chen et al. developed an approach called IntelligentCrowd that leverages the power of multi-agent reinforcement learning (MARL) to address the problem of task allocation [18]. Cen et al. introduced a stochastic task recommendation framework that takes into account workers’ historical trajectories, preferred time budgets, and the inherent probabilistic uncertainty of their future trajectories [19]. In addition to these studies, many user-centric task allocation approaches also prioritize aligning their objectives with the protection of participant privacy [20,21,22,23,24,25,26]. A key privacy concern with user-centric approaches lies in their reliance on gathering extensive user data, which increases the risk of privacy breaches [6].

Some works have tackled incorporating fairness into task allocation solutions.Song et al. developed the Fairness Task Assignment Strategy with Distance Constraint (FTAS-D), which leverages Lyapunov optimization to identify a feasible assignment solution and iteratively refines it to find the optimal one [30]. This approach effectively balances the objectives of minimizing costs while ensuring fairness in task distribution among users. In this context, fairness in task allocation involves preventing any single user from being overloaded by distributing tasks evenly to avoid unfair burdens. It also means ensuring that each task is assigned an appropriate frequency so that no task is neglected or favored disproportionately. Duan et al. developed a task allocation procedure that incorporates a fairness-aware policy [31]. Their approach focuses on fairness related to the contribution each participant makes to task performance, rather than on task distribution or the effort required to complete the tasks. Li et al. introduced incentive mechanisms for task allocation that prioritize both fairness and energy efficiency [32]. In terms of fairness, the incentives are determined by evaluating the participants’ performance.

The solutions mentioned above are designed to achieve an equilibrium between fulfilling their objectives and being economically advantageous, maximizing the platform’s profit, maximizing the economic reward for users, and/or minimizing transportation costs, among other possibilities. That is, all of these solutions include an economic optimization component. However, many applications, such as traffic, noise, and weather monitoring, typically do not offer financial rewards to users involved [28]. To the best of our knowledge, only one author has examined task allocation within the context of altruistic and unpaid participation over the past five years. Meitei et al. have developed several approaches in which they eliminated profit-driven motives [33,34,35]. In their first work, they proposed two user-oriented task allocation schemes: Nearest User Task Allocation (NUTA) and Nearest User Fair Task Allocation (NUFTA). They focused on achieving an optimized and equitable workload for users [33]. Following this work, they refined their initial two algorithms and introduced two additional user-oriented solutions to accommodate time and distance constraints: Time-Sensitive Task Allocation (TSTAS) and Time-Sensitive Task Allocation with Time Slot Change (TSTA_TSC) [34]. The main goals of these approaches were to (1) minimize the traveling distance between users and their tasks and (2) allocate as many tasks as possible while satisfying time-dependent conditions. Their most recent work introduced a new multi-objective, time-dependent task allocation method called Load-Balanced Task Allocation (LBTA) [35]. The primary objectives of this approach are to (1) allocate as many tasks as possible and (2) create a load-balanced distribution of tasks during the allocation.

However, other challenges related to altruistic participation must be addressed, such as the possible decrease in participation or the overloading of users. Consequently, there is significant potential for developing task allocation strategies that prioritize enhancing the user experience, thereby fostering more sustained and effective participation that is not solely based on financial rewards.

## 3. Problem Formulation

In this section, the time and spatial constraints of the task allocation problem are formulated. This solution can be applied to multiple SC problems. As part of our goal, this formulation must integrate a workload limitation to improve the user experience and prevent overloading. The variables used in our problem formulation are listed in Table 1.

**Figure 1:**
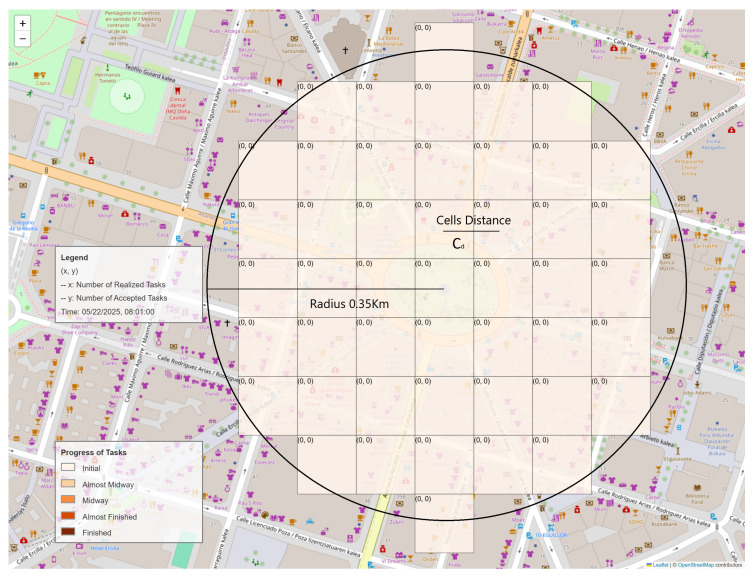
An example of a campaign created in Bilbao is presented. In this picture, the distribution of the cells and the color scale implemented based on the $M_{s}$ goal of the campaign are shown. Given the nomenclature defined in this paper, x = $rea_{j}^{t}$, and y = $accep_{j}^{t}$.

The task allocation procedure is referred to as a campaign. In this work, the spatial component is defined as a circular area centered at a specific point with a given radius. Alternative geometric shapes could be considered in the future. This area is further subdivided into a grid of squares, called cells, for task distribution. This subdivision is based on the input parameter $C_{d}$, which defines the distance between the centers of two adjacent cells. Using this parameter and the defined circular area, the grid is generated. Each campaign contains at least one cell within the entire campaign area. The cells remain consistent throughout the campaign since it is a static problem. The total number of cells in a campaign is denoted as *m*, and the *j*-th cell is represented as $L_{j}$. Let *L* denote the set of all cells within a campaign (Equation (1)). Figure 1 illustrates the spatial component of a campaign and how the cells are distributed within this area. Tasks must be performed at the center of these cells.

The campaign’s temporal component is divided into sub-periods called slots. All campaigns always have at least one slot. The number of slots is calculated based on the input $d_{s}$, which defines the slot duration. Hence, the duration of one campaign is divided into sub-periods of equal length, except for the last slot, which may span a shorter duration. Based on this input, the number of slots is defined and denoted as *n*. The *i*-th slot is described as $S_{i}$, and *S* is the set composed of all the campaign slots (Equation (2)). The following equations show the formulation of the sets *L* and *S*, where *L* is composed of the campaign cells, and *S* is composed of the campaign slots.

(1) $$\begin{array}{cc} L=\; & \{L_{0},\cdots ,L_{m-1}\};\;\;\;\;\;m\geq 1 \end{array}$$

(2) $$\begin{array}{cc} S=\; & \{S_{0},\cdots ,S_{n-1}\};\;\;\;\;\;n\geq 1 \end{array}$$

As this is a time-dependent problem, each slot, $S_{i}$, is associated with two times: the time it starts, $St(S_{i})$, and the time it ends, $Ft(S_{i})$. Based on this notation, the start of the campaign can be expressed as $St(S_{0})$ and the final time as $Ft(S_{n-1})$. The problem must satisfy three constraints: (Equation (3)) the start time of a slot must occur before its end time; (Equation (4)) the duration of all slots is the same, $d_{s}$, except for the last slot, which may be shorter; and (Equation (5)) the ending time of one slot must coincide with the beginning time of the next slot.

(3) $$\begin{array}{cc} St\left(S_{i}\right)<Ft\left(S_{i}\right);\;\;\;\;\;\;\;\;\;\;\;\;\;\;\;\;\;\;\;\;\;\;\;\;\; & \;\;\;0\geq i\geq n-1; \end{array}$$

(4) $$\begin{array}{cc} Ft\left(S_{i}\right)-St\left(S_{i}\right)=Ft\left(S_{0}\right)-St\left(S_{0}\right)=d_{s}; & \;\;\;0\geq i\geq n-2; \end{array}$$

(5) $$\begin{array}{cc} Ft\left(S_{i}\right)=St\left(S_{i+1}\right);\;\;\;\;\;\;\;\;\;\;\;\;\;\;\;\;\;\;\;\;\;\; & \;\;\;0\geq i\geq n-2; \end{array}$$

Limiting the workload is paramount to enhancing the user experience. Since participation is altruistic, avoiding the overloading of users is essential to sustaining engagement. Hence, in this work, each campaign is associated with a fixed number that defines the maximum number of allowed completed tasks in each cell at each slot, denoted as $M_{s}$. The variable $M_{s}$ depends on the problem and its specific requirements. When the task involves capturing information by a sensor, multiple sensor measurements or tasks are needed to ensure that the average value is representative, as the sensor readings can vary based on measurement conditions. If the tasks are unrelated to sensor measurements, it may be sufficient to perform only one task. The idea is to use the $M_{s}$ tasks of each cell to obtain a representative value for the cell in this slot. These representative values compose the final data time series map. Consequently, only $M_{s}$ tasks can be realized at each location of *L* for each slot, $S_{i}$. Thus, a task can be represented as $t_{j,i}^{k}$, which is the *k*-th task of cell $L_{j}$ at the *i*-th slot $S_{i}$. The set of all tasks of the campaign can be expressed in Equation (6). The total set of tasks defined by this equation outlines the task completion requirements and goals of the campaign, representing the task completion effort users must undertake to complete the dataset.

(6) $$\begin{array}{c} {\left\{t_{j,S_{i}}^{0},\cdots ,t_{j,S_{i}}^{M_{s}-1}\right\}}_{j\in L};\;\;\;M_{s}\geq 1 \end{array}$$

A color scale was designed to facilitate the visualization of the campaign’s progress towards the task completion goal, with the maximum number of completed tasks allowed defined as $M_{s}$. As shown in Figure 1, the color scale consists of five distinct colors. In this example, $M_{s}$ is set to eight tasks.

In conclusion, this section has defined and modeled the spatial crowdsourcing problem in the context of citizen science. The parameters defined, regarding both spatial conditions (number of cells, $C_{d}$, and others) and temporal conditions of the campaign (number of slots, n), as well as the maximum number of tasks desired per cell ($M_{s}$), must be established according to the specific needs of the citizen science problem at hand and the expected number of participants. Since citizen science projects are highly varied, the characteristics of these campaigns must be adaptable to different contexts. Therefore, this section describes a model with these features, capable of adapting to various citizen science contexts.

## 4. Volunteer Task Allocation Engine

This section explains the task allocation engine called the Volunteer Task Allocation Engine (VTAE). The solution must associate an available user with a spatial task and be user-centric, allowing users to choose tasks from a range of recommendations and thereby enhancing the user experience. Hence, when a user wants to participate, they receive a given number of tasks and must select which ones they wish to complete. Currently, the system is configured to recommend a maximum of three tasks for each request. In future works, the number of tasks suggested to users can be configured. Additionally, the solution must ensure a balanced task distribution across static locations. As mentioned, one representative value is calculated from the $M_{s}$ tasks completed in a specific cell and slot. The set of representative values composes the final data map of the campaign. Ensuring an equal distribution of tasks across locations is crucial because representative values must be derived from a comparable number of samples (tasks). If one representative value is based on a small sample size and another on a large one, they become statistically incomparable. Furthermore, participation is altruistic and not guaranteed, so it can abruptly decrease. Therefore, maintaining an equal distribution of tasks across locations is a key consideration for task allocation in the context of altruistic participation. Hence, the VTAE must ensure that the distribution of tasks is uniform with respect to cells, taking into account that users must choose the task they want to perform from those recommended.

To achieve this, the first step is to create a system for managing uncompleted tasks. When a user promises to complete a task and does not do so, the system must assign this task to another person. Without this, the task cannot be completed, and the task completion objective cannot be achieved. For this reason, each recommendation, that is, the tasks that the system suggests to the user, is associated with a status. The possible statuses are as follows: *”notified”* if the task has been recommended to the user, *”accepted”* if the user agrees to perform the task, *”realized”* if the user completes it, and *”not realized”* if the user does not complete it. To achieve the state of ”not realized”, the status of the recommendation must be ”notified” or ”accepted” within a few minutes; after that time, this status changes to ”not realized” automatically, and the task can be assigned to another user.

In addition, to achieve a uniform distribution, a priority system was created to ensure this balance. It describes the cell’s priority, indicating how much the cell needs a task. This metric considers several campaign features, such as the remaining time until the slot expires and the number of completed and accepted tasks in the cell. By comparing this metric across various cells, it can be determined which cell has a greater number of realized tasks, thereby creating a uniform distribution of tasks across all cells. Let *t* be the current time, $S_{i}$ the slot corresponding to *t*, $d_{s}$ the span of the slots, $rea_{j}^{t}$ the number of realized tasks of the cell $L_{j}$ in the slot $S_{i}$, $accep_{j}^{t}$ the accepted tasks of the cell $L_{j}$ in the slot $S_{i}$, and $M_{s}$ the maximum number of completed tasks allowed per cell. Then, the priority of the cell $L_{j}$ at time *t* can be denoted as $P_{j}^{t}$ and is defined in Equation (7). This metric facilitates efficient task management by dynamically adjusting task priorities based on real-time demand and user engagement. An automated scheduled process assesses the priority of cells in the campaign every few minutes. This means that the VTAE always has access to up-to-date priority values for each cell, eliminating the need to recalculate them every time a recommendation is requested. All the notations and symbols are explained in Table 1.

(7) $$\begin{array}{c} P_{j}^{t}=\frac{\left(t-St\left(S_{i}\right)\right)}{d_{s}}-\frac{accep_{j}^{t}+rea_{j}^{t}}{M_{s}}\\ j\in \{0,\cdots ,m-1\};\;\;i\in \{0,\cdots ,n-1\} \end{array}$$

One of the primary objectives is to enhance the user experience by adopting a user-centric approach. In this approach, users will be presented with a list of up to three tasks, and they are required to select one to complete. The selection of recommended tasks for this list is determined by the priority of the cell and the user’s location. Furthermore, to adapt the recommendations to the users and avoid overloading them, only cells close to the user are considered for recommendation. In this manner, the user is not required to travel far to complete the task. A cell is deemed to be in proximity to the user if, and only if, the distance between the cell center and the user’s position is less than five times the $C_{d}$ distance. Other alternative definitions of close cells can be configured in future versions, such as defining a maximum distance. The initial step in generating task recommendations involves identifying all closer cells to the user. To create the task recommendations for each of these cells, priority, the number of accepted and completed tasks, and the distance from the user are calculated. The list of cells is then ordered by fewer accepted plus completed tasks, higher priority, and closer proximity. The top three cells of this ordered list are recommended to the user.

**Figure 2:**
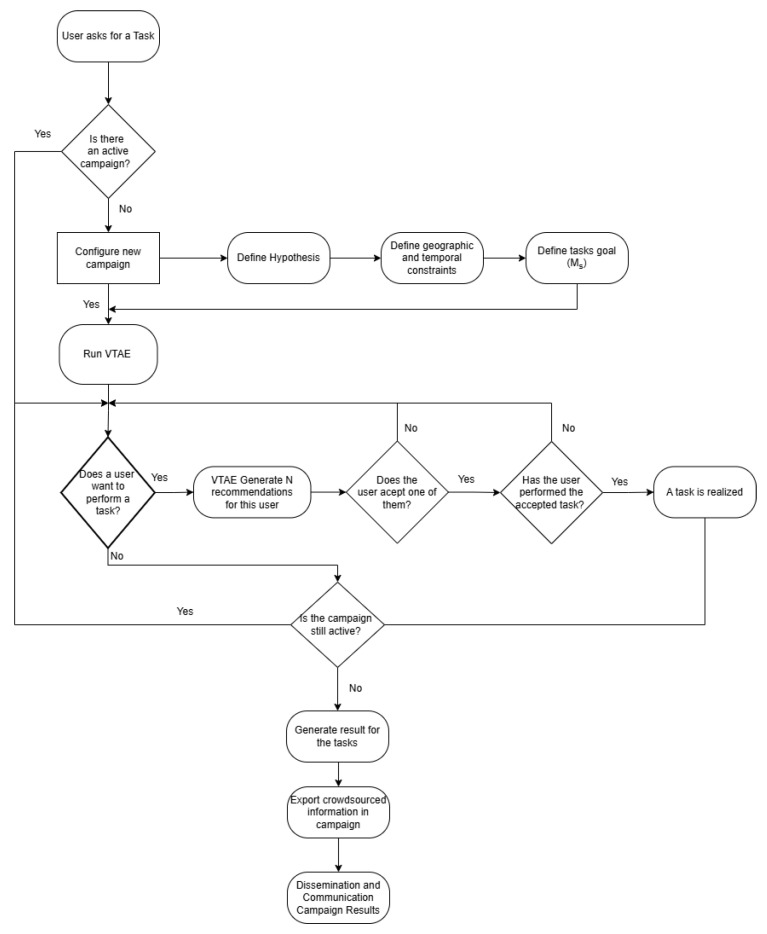
Workflow illustrating the interaction between the VTAE system and users during a campaign. The process includes key steps such as the campaign configuration, task recommendation, user participation, task completion, and dissemination of results in the context of Citizen Science.

To illustrate how the VTAE interacts with users and the general workflow of a campaign, a diagram has been designed that outlines the key steps in the process, as shown in Figure 2. The interaction begins when a user wants to participate in a task. For this to be possible, there must be an active campaign. If there is no ongoing campaign, a new one must be configured by defining the hypothesis, the geographical area, temporal constraints, and the specific task goals, as detailed in Section 3. With an active campaign, the VTAE generates three specific task recommendations for the user. The user must select one of the suggested tasks and proceed to complete it. Once the task is successfully completed, the VTAE continues assigning tasks to other users interested in participating, maintaining the interaction cycle between the system and the users while the campaign remains active. When the campaign concludes, the results obtained from all completed tasks are generated and analyzed. Since this process takes place within a citizen science context, the results must be disseminated and communicated, ensuring their value for the defined social benefit.

In future versions, the shape of the campaign, the definition of the close cells, and the number of tasks that the VTAE recommends to the user will be configurable variables. This solution, called VTAE, is implemented in an API found on GitHub at https://github.com/mpuerta004/RecommenderSystem, (accessed on 15 December 2024) (This repository encompasses multiple developments, not all of them related to the VTAE. However, both the simulation presented in Section 6 and the development of the VTAE can be found in this repository). This solution associates an available user with a task independently of the type of task or the spatial crowdsourcing problem.

## 5. Use Cases Details and Evaluation Metrics

This section evaluates the efficiency and performance of the VTAE in addressing the proposed goals in the context of altruistic participation. The VTAE’s first goal is to increase the user experience by limiting the workload and creating a user-centric resolution. The second goal is to create an equal distribution of tasks over static locations, achieving the maximum task completion possible without exceeding the campaign threshold.

The performance metrics used to evaluate the VTAE are given below:

- **Variance:** Measure the equal distribution of the tasks over the locations. Low variance denotes an even distribution of tasks across various locations, whereas high variance signifies an uneven distribution, leading to inequality datasets.
  (8) $$Var^{t}=Var\left({\left\{rea_{j}^{t}\right\}}_{j\in L}\right)=\frac{\sum_{j\in L}{\left(rea_{j}^{t}-\bar{rea_{j}^{t}}\right)}^{2}}{n-1}$$
- **Task completion ratio (TCR):** Measure the ratio of tasks realized versus the total number of tasks that the campaign defined based on the $M_{s}$ and the number of cells. A higher TCR represents a higher percentage of tasks performed. This metric is calculated at the end of each slot.
  (9) $$TCR=\frac{\sum_{j\in L}\sum_{S_{i}\in S}rea_{j}^{Ft(S_{i})}}{m*M_{s}}$$
- **Evolution of the realized tasks over time:** The number of realized tasks over time. This metric allows us to understand which task allocation algorithm has more realized tasks over time. In this case, the temporal series is based on the number of tasks realized each minute of the campaign.
  (10) $$\{\sum_{j\in L}{\left(rea_{j}^{t}-rea_{j}^{t-1}\right\}}_{t}$$

In addition, two baseline algorithms were developed to assess the effectiveness of the VTAE. The first baseline, called the near neighbors (NN) algorithm, assigns a user to the closest cell to perform a task. The second baseline, the limited near neighbors (LNN) algorithm, also assigns a user to the nearest cell but includes the same workload limitation as the VTAE. Specifically, in the LNN algorithm, each cell has the same predefined threshold for the workload $M_{s}$ as defined in the campaign.

VTAE was tested and compared to the baselines using two approaches: a computer simulation of the task allocation problem and two pilot studies. The simulation evaluates the system’s performance under controlled conditions, while the pilot study assesses the system’s real-world applicability with real users. In conclusion, the assessment of the VTAE solution relies on the following three experiments:

- **Simulated demo:** This is a simulation of the task allocation problem designed to ensure the accurate behavior and performance of the algorithm under diverse conditions.
- **Deusto experiments:** It encompasses a focused, small-scale (12 participants) trial carried out at our University of Deusto. This experiment aimed to create a collage of photos representing users’ vision of the university and their surroundings. This pilot allowed us to evaluate the effectiveness of VTAE for altruistic participation in a real experiment and evaluate user comfort and acceptance through a quantitative evaluation.

In the following sections, these experiments are explained.

## 6. Simulated Demo

This simulation models the task allocation problem to evaluate the effectiveness and regulation of the distribution of the VTAE. It replicates user behavior in the context of altruistic participation, where users follow their daily routines. The system accounts for various behaviors, ranging from predictable routines to complete randomness.

At the start of the simulation, each user is assigned an initial trajectory that represents their daily routine, along with a probability reflecting how likely they are to repeat this pattern. This probability defines the rigidity of their adherence to the routine. A user’s position is updated based on their last known location and trajectory whenever they become available. It is assumed that, during periods of unavailability, users travel a random distance toward their target. The trajectory concludes once the user reaches the predefined endpoint; if they do not repeat the original one, a new random trajectory is generated. The simulated user either accepts or rejects task recommendations based on how well they align with their current trajectory or proximity. When a user accepts a recommendation, a random time is assigned to simulate the duration required to reach the task location.

Simulation experiments are based on three customized variables:

- **Number of users:** This parameter defines the number of participants in the simulation. Once the task completion requirements are established, the individual effort required from each user can be evaluated. With a larger number of users, the effort per user decreases, assuming the task completion requirements remain constant. Conversely, with fewer users, the likelihood of failing to meet the task completion objectives increases, making efficient task distribution essential. Varying the number of users serves as a test to assess the performance of the VTAE under different levels of user effort.
- **User task completion probability:** This indicates the likelihood of a user completing an accepted task. When a user accepts a task, a random time is generated to determine how long it will take to reach the task location. Once this time has elapsed, the completion probability determines whether the user successfully completes the task. High task completion signifies a considerable commitment from users to their accepted tasks. Experiments with different task completion probabilities were conducted to evaluate the effectiveness of the VTAE against varying levels of user commitment.
- **User availability probability:** This parameter represents the probability of a user being available to request a task at any moment. It models the likelihood of a user requesting a task, not necessarily accepting one. Users are considered unavailable if they have pending tasks. In simulations with high user availability, this reflects a strong motivation to participate, leading to an increase in completed tasks. Experiments with different availability probabilities were conducted to evaluate the effectiveness of the VTAE under varying levels of user motivation.

Using this simulation, several experiments were conducted to analyze the performance of the VTAE. These experiments maintained the campaign configuration while varying the customized variables to assess their impact and behavior. The campaign configuration for all the experiments included two slots, each lasting 2 h. The campaign was located in the center of Bilbao at a longitude of −2.9349889768009008 and a latitude of 43.26302147532318, with a radius of 0.2 km. The campaign started on 22 May 2024 at 08:00:00 and ended on the same day at 12:00:00. The $C_{d}$ was 0.05 km, and $M_{s}$ was eight tasks. A visualization of this campaign is shown in Figure 1. The following subsections explain the experiments based on these three variables.

### 6.1. Experiment 1: Crowd Size Variation

This experiment evaluates the effectiveness of the VTAE under different levels of user effort. An increase in the number of users for the same task completion effort reduces the individual effort required, as each user contributes less to meeting the overall requirements. Conversely, when fewer users are involved, the effort per user increases, making it more challenging to achieve the task completion goals. In such cases, balanced task distribution becomes critical for successfully generating a complete and accurate data map. In this experiment, various levels of user effort were tested while fixing the task completion efforts, creating four campaigns with 100, 75, 50, and 25 users. With 100 users, individual effort is minimal, whereas with 25 users, the effort required is significantly higher. In these campaigns, the task completion probability variable is fixed at 75%, indicating a strong commitment from users to the tasks they accept, while the user availability probability is set to 50%. The VTAE, LNN, and NN algorithms were tested with the different user counts to compare the results. Five simulations were conducted for each user count and task allocation algorithm, with the results presented as the mean values of these five executions.

**Figure 3:**
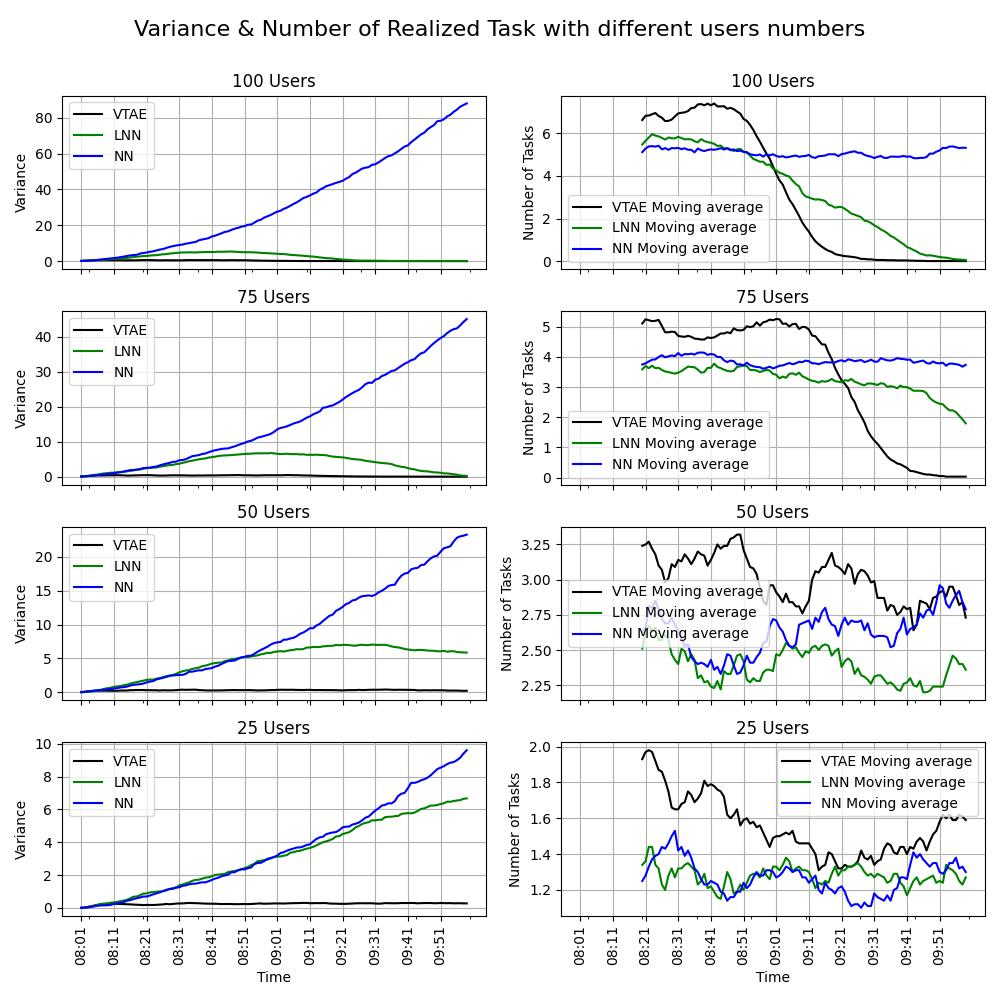
The variance in the distribution of the tasks over the cells (Equation (8) left hand-side pictures) and the evolution of the realized task (Equation (10) right hand-side figures) over time across different users count (100, 75, 50, and 25 users). The task allocation algorithms used are the VTAE, LNN, and NN. To illustrate the evolution of the task realized, the moving average (MA) of 20 values was used. The results presented are the mean of five executions.

The variance (Equation (8)) and the evolution of the tasks realized over time (Equation (10)) for the four campaigns with the three algorithms are shown in Figure 3. Since the campaign consists of two slots and the task completion objectives were reset at the beginning of each slot, only the first slot is presented. Additionally, the moving average of this time series is included to simplify the visualization of the evolution of the tasks realized. The task completion rate (TCR) metric for the campaigns is presented in Table 2.

The table shows that, as the number of users increases, the task completion ratio (TCR) rises across all algorithms. Additionally, in both the VTAE and LNN algorithms, which impose a workload limitation, the TCR does not exceed 100%. This expected outcome confirms the proper implementation of the workload limitation, preventing users from becoming overloaded. In contrast, the NN algorithm exceeds this limitation when 100 and 75 users are involved, as it lacks any workload restrictions. However, the task distribution is unbalanced (as shown in the left images of Figure 3), exhibiting an exponential pattern. This imbalance renders the final map statistically irrelevant. A similar variance pattern is observed in the LNN algorithm with 25 users, which is expected since both baselines are similar, although LNN imposes a workload limitation. The VTAE, on the other hand, demonstrates lower variance, highlighting its effectiveness in ensuring a more even distribution of tasks across static locations.

Moreover, in scenarios where the NN algorithm does not exceed the workload limitation, the VTAE consistently achieves a higher task completion ratio (TCR) (Table 2). This indicates that, without the imposed limitation, the VTAE would outperform the NN in terms of TCR. Furthermore, the TCR of the LNN is always equal to or lower than that of the VTAE. This is further corroborated by the right-hand side of Figure 3, where the moving average of realized tasks is consistently higher for the VTAE. The only exception occurs near the end of the slot in the 100 and 75 user cases, where the VTAE’s performance declines sharply. This decline happens because the task completion requirements have been met, leading users to stop receiving tasks as a deliberate mechanism to prevent overloading. Consequently, the number of realized tasks decreases due to the limitation on task-completion efforts.

In conclusion, the VTAE effectively demonstrates the efficiency of its task allocation approach. The algorithm adheres to the task execution limitation set by the campaign, ensuring that users are not overloaded. Additionally, in scenarios where user effort is high but task completion requirements are not fully met, the VTAE achieves an equitable distribution of tasks across locations, resulting in low variance. This is a key objective that the NN resolution fails to achieve. Furthermore, the task completion ratio (TCR) consistently indicates that the number of tasks completed with the VTAE exceeds that of the LNN in all scenarios. Overall, the VTAE outperforms both the NN and LNN algorithms across varying levels of user effort.

### 6.2. Experiment 2: User Availability Probability

In this experiment, different availability probabilities were tested to evaluate the effectiveness of the VTAE under varying levels of user motivation. Campaigns with high availability indicate motivated users, facilitating the achievement of task completion goals. Conversely, when user availability is low, both involvement and motivation decrease, making it more challenging to meet the task completion requirements. In such cases, the task allocation algorithm must ensure an even distribution of tasks across static locations to produce a relevant data map. Availability probabilities of 75%, 50%, and 25% were tested. The algorithms compared were VTAE, LNN, and NN, with 50 users and a fixed task completion probability of 75%. The results presented are the averages of five executions.

**Figure 4:**
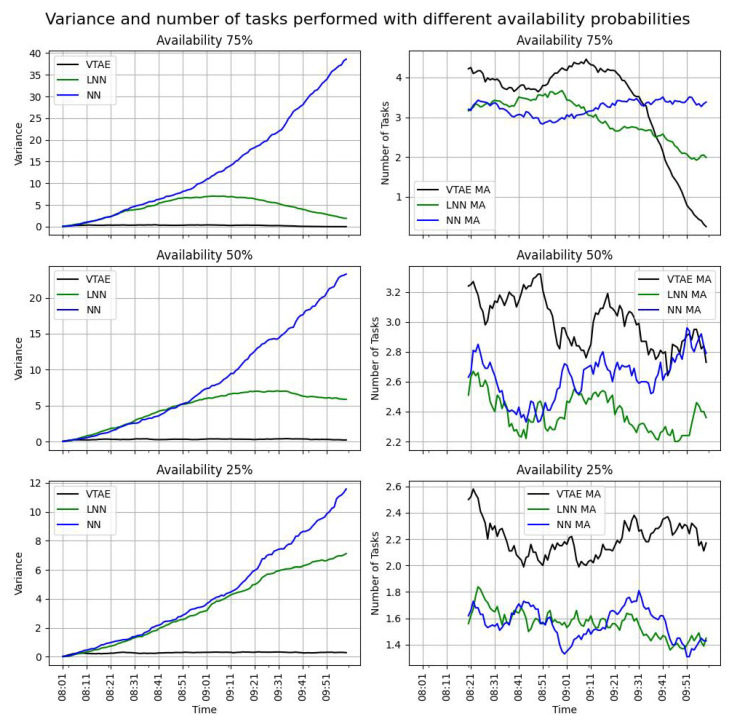
The variance (Equation (8) left figures) and the evolution of the realized task (Equation (10) right figures) over time across different user availability probabilities. The task allocation resolutions used are the VTAE, the LNN, and the NN algorithms. To illustrate the evolution of the task realized, the moving average (MA) of 20 values was used. The results show the mean of five executions.

The variance (Equation (8)) and the evolution of tasks realized over time (Equation (10)) for these three campaigns are shown in Figure 4 (left and right columns, respectively). Since each campaign consists of two slots and the task completion objectives were reset at the beginning of each slot, only the first slot of each campaign is displayed in Figure 4. The task completion ratio (TCR) metric can be observed in Table 3.

This table illustrates the effect of the workload limitation at 75% availability, as the VTAE restricts users to a maximum of eight tasks per cell in each slot. Consequently, no more than 100% of the task completion target can be achieved. The NN algorithm outperforms the VTAE in the task completion ratio (TCR) metric only in this scenario, as it does not impose a workload limitation. In all other availability percentages, the VTAE significantly exceeds the TCR of both the NN and LNN algorithms. This trend is further supported by the right column of Figure 4, which shows that the number of realized tasks for the VTAE consistently surpasses that of the NN and LNN. The only exception occurs at the end of the 75% scenario, where the realized tasks for the VTAE decline due to the workload limitation established by the campaign. In the left column of the figures, the variance in the NN exhibits an exponential pattern. Initially, the LNN demonstrates similar behavior due to the similarities between the two algorithms. However, the limit imposed by the LNN results in a decrease in variance. In contrast, the VTAE maintains a low variance throughout the slot, demonstrating its effectiveness in ensuring an equal distribution of tasks across static locations.

Based on these results, it can be concluded that user motivation is crucial for achieving optimal task completion outcomes. Moreover, even with low availability probabilities, the VTAE demonstrates superior performance by adhering to the limits set by the task completion campaign and ensuring a balanced distribution of tasks across the cells, resulting in a higher task completion ratio (TCR).

### 6.3. Experiment 3: User Task Completion Probability

In this experiment, the task completion probability variable was tested to understand how user engagement with their accepted tasks affects the effectiveness of the VTAE. A high task completion probability indicates a strong user commitment and leads to a greater number of realized tasks by the end of the campaigns. This section evaluates task completion probabilities of 75%, 50%, and 25%. The algorithms used were VTAE, LNN, and NN, with the number of users set to 50, and the user availability probability was set to 50%. The results presented are the means of five executions.

**Figure 5:**
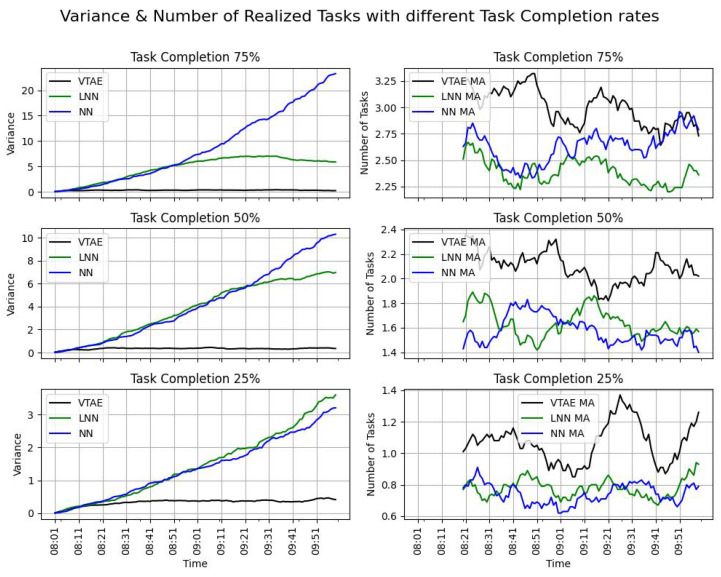
The variance of the distribution of the tasks over the cells (Equation (8)—left figures) and the evolution of the realized task (Equation (10)—right figures) over time across different task completion probabilities. The task allocation resolutions used are the VTAE, the LNN, and the NN algorithms. To illustrate the evolution of the task realized, the moving average of 20 values was used. The results show the mean of five executions.

The variance (Equation (8)) and the evolution of tasks completed over time (Equation (10)) are presented in Figure 5 (left and right columns, respectively). Since the campaign consists of two slots, and the task completion objectives are reset at the beginning of each slot, only the results from the first slot are displayed in the figures. The task completion rate (TCR) metric is provided in Table 4.

As in the previous experiments, the number of tasks completed using the VTAE (shown in the right column of Figure 5) is consistently higher than that of the other algorithms. This indicates that more tasks are performed with this algorithm, leading to a higher task completion rate (TCR) in all cases, as demonstrated in Table 4. In this experiment, the workload limitation was not reached for any of the tested values; thus, as observed in previous experiments, the TCR for the VTAE algorithm remains higher, along with the number of tasks completed.

Regarding the variance, a similar pattern is observed: the variance for the VTAE is low. In contrast, the NN exhibits exponential behavior in terms of variance, which begins to decrease, as it cannot exceed the workload limit of eight tasks per cell in each slot.

It is noteworthy that, as the availability percentage varies, the TCR of the VTAE ranges from 100% to approximately 61.38%. Conversely, as the task completion rate varies, the TCR of the VTAE ranges from 95.43% to 31.33%. Consequently, it can be inferred that the task completion rate variable exerts a greater influence on the behavior of the VTAE in accomplishing the task completion objective.

In conclusion, the VTAE ensures an equitable distribution of tasks across spatial locations. It achieves a higher number of realized tasks in all campaigns and consistently surpasses the task completion rate (TCR) attained by both the LNN and NN algorithms.

### 6.4. Conclusions of the Simulated Demo Experiments

In this section, the VTAE was evaluated through a simulation designed to replicate user behavior in the context of spatial crowd sensing and altruistic participation. The results indicate that the VTAE effectively maintained low variance, ensuring an equitable distribution of tasks across static locations.

The NN algorithm surpassed the task completion rate (TCR) of the VTAE only in scenarios where the VTAE’s performance decreased due to workload limitation. In cases where the workload limitation was not reached and user activity persisted, the VTAE consistently outperformed the NN. Furthermore, the NN exhibited an exponential pattern in the variance, resulting in certain cells receiving significantly more tasks than others. This uneven distribution compromises data integrity and validity, as it risks overburdening users by exceeding the workload limitation, which in turn leads to decreased data quality. In contrast, the LNN consistently achieved a TCR that was equal to or lower than that of the VTAE. Although the LNN’s variance was less pronounced than that of the NN, it remained considerably higher than that of the VTAE.

In conclusion, the VTAE demonstrates superior overall performance, consistently achieving reliable results across various parameters. This underscores its robustness and reliability, confirming its ability to meet the objectives set for this task allocation problem.

## 7. Deusto Experiment

This section evaluates the VTAE using a real-world scenario involving actual users from the University of Deusto (https://www.deusto.es/en/home, accessed on 15 December 2024). In this experiment, participants were tasked with capturing photos of the University of Deusto to create a collage. The VTAE distributed the tasks (taking pictures) among the participants. As the VTAE algorithm is agnostic to the spatial crowdsourcing problem, this experiment serves as a practical example of such a problem, allowing for the assessment of the VTAE’s effectiveness in a controlled, real-world context.

For this pilot, the VTAE’s API was connected to a Telegram (https://web.telegram.org/a/, accessed on 15 December 2024) bot specifically developed to interact directly with users. Both the bot and the VTAE are available in the GitHub repository at https://github.com/mpuerta004/RecommenderSystem, (accessed on 15 December 2024).

For this experiment, 12 participants were recruited via a convenience sampling method from the Deusto University staff. Each participant booked two hours of time, during which they were shown a training presentation lasting a maximum of thirty minutes. The remainder of the time was allocated to conducting the experiment. During the presentation, the goals of the experiment and the functionality of the Telegram bot were explained. Upon completion of the training, the participants began the experiment.

**Figure 6:**
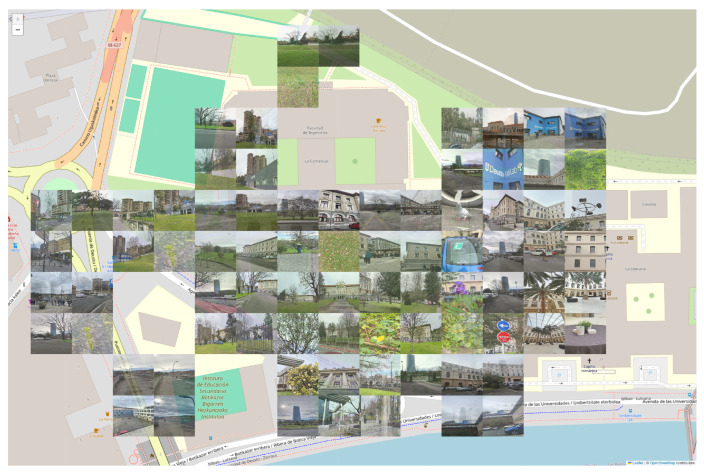
The final collage of photos obtained from the Deusto experiment.

The experiment took place on 23 February 2024, from 10:00:00 to 12:00:00, centered at the University of Deusto (longitude: −2.9396777560573013, latitude: 43.271157163906985). The parameter $C_{d}$ was set to 0.05 km, and the campaign’s radius was 0.18 km. Additionally, several cells were removed due to inaccessibility. These characteristics were chosen to ensure an appropriate number of cells, allowing users to move comfortably from one cell to another without excessive effort while also ensuring an efficient distribution, considering the eliminated cells as well. Additionally, a value of $M_{s}$ equal to four tasks was selected so that users could complete the tasks quickly and easily within the established time. The cell distribution is shown in Figure 6, which also displays the final collage. The pilot concluded that, when users stopped receiving recommendations, it was either because the task completion goal had been achieved or the allotted time had expired.

The VTAE aimed to enhance the user experience, and its evaluation was based on qualitative and quantitative data. After the experiment, users were asked to complete a survey to assess their user experience and perception of the VTAE. The following section provides the qualitative and quantitative evaluation of the VTAE in the Deusto experiment. The quantitative findings are based on data collected during the experiment, while the qualitative results are gathered from a user survey.

### 7.1. Quantitative Results

The task allocation process commenced at 10:33 and concluded at 11:15, as users no longer received recommendations. They achieved the experiment’s task completion goal before the campaign ended. Based on the variable $M_{s}$, the objective was to obtain 84 images; this goal was exceeded, resulting in a total of 92 photos collected. Two factors contributed to this outcome: (1) When a recommendation is generated, another participant may have accepted it beforehand, creating the potential to exceed the workload limitation. (2) The system accepts photos taken in incorrect locations while requesting that they be captured in the designated areas. This flexibility helps prevent user frustration, particularly when some cells may be inaccessible. Of the total, 46 photos were attributed to this second reason, largely due to the small size of the cells and issues with users’ GPS accuracy. These factors are illustrated in the final collage (Figure 6), where one cell in the top left corner contains only three photos. The location of the cell’s center complicates taking a picture within its boundaries, as nearby locations can be easily associated with other cells. While these reasons facilitated participants in exceeding the limit, it is important to note that, even though the workload limitation was surpassed, the overall effort remained limited.

**Figure 7:**
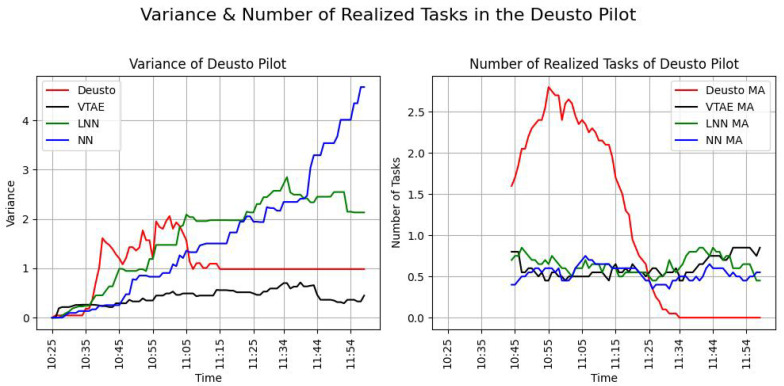
Evolution of the variance (metric of Equation (8)—left hand-side picture) and realized tasks over time (Equation (10)—right hand-side picture) in the Deusto experiment and the result obtained in its simulation using the VTAE, LNN, and NN. To illustrate the evolution of the task realized, the moving average (MA) of 20 values was used.

To compare the effectiveness of this experiment with the simulated one, a simulation of this campaign was recreated. The baselines (LNN and NN) and the VTAE were used for this proposal. The simulation was configured with a 50% task completion rate and a 75% task completion probability, with twelve users simulated to resemble the real data as closely as possible. Figure 7 illustrates the variance and the evolution of the realized tasks throughout the experiment (left and right panels, respectively). Table 5 presents the task completion ratio (TCR) metric for this campaign using the three algorithms and the data for the real one. In this Table, the mean and standard deviation (SD) of the variance metric (Equation (8)) and the number of tasks completed per minute (Equation (10)) can be shown. These metrics were added to provide a clearer comparison of the differences and similarities between the simulated and real-world performance of the VTAE.

Figure 7 on the left-hand side represents the defined metric of the variance (Equation (8)) in task distribution over time. In the real-world Deusto Pilot, represented by the red line, the variance remained relatively low and stable (mean variance ± SD variance is equal to 1.03 ± 0.23), indicating that the distribution of tasks among participants was well balanced. Among the simulated scenarios, the VTAE (black line) achieved the lowest variance (mean ± SD is equal to 0.42 ± 0.02). Table 5 confirms that the VTAE outperformed other algorithms, achieving the lowest mean variance and SD variance. The LNN algorithm (green line) initially exhibited low variance but experienced a significant increase over time, indicating difficulties in maintaining balance as time progresses. This trend is also evident in Table 5, where the mean variance of the LNN algorithm is higher compared to other algorithms. Although its standard deviation (SD) is smaller than that of other algorithms, the mean variance of 1.64 highlights notable variability, albeit with fewer fluctuations, as indicated by its SD of 0.72. In contrast, the NN algorithm also has a mean variance of 1.64 but shows significantly greater variability, with a much higher SD of 1.62.

The right-hand side of Figure 7 illustrates the number of realized tasks (tasks completed) over time. In the real world, represented by the red line, the number of tasks increases sharply at the beginning, peaking around 11:00 before declining because the users are finishing the campaign. This pattern indicates a saturation point at which most tasks were completed. This saturation and the sharp peak are also reflected in the mean of the metric and its standard deviation, as shown in Table 5. The mean number of tasks completed per minute is 0.97, with a standard deviation of 2.42. These numbers indicate that it is the algorithm that completes the most tasks per minute, but it also exhibits the highest fluctuation observed among the four analyzed algorithms. In the simulated scenarios, the VTAE (black line) shows a consistent and steady completion of tasks, mirroring real-world dynamics more effectively than the baseline algorithms. The LNN algorithm (green line) also performs moderately well but exhibits more fluctuations, which suggests some inconsistency. In Table 5, the LNN has a lower mean of tasks completed per minute and less variance than the VTAE, with a mean of 0.61 and a standard deviation of 0.58. Lastly, the NN algorithm (blue line) lags behind the other simulations, indicating a lower task completion rate and less effective task allocation. These insights from the picture are reflected in Table 5, where the NN algorithm has a mean of tasks completed per minute of 0.51 and a standard deviation of 0.47. These are the lowest mean and SD values observed in the experiment. The mean and standard deviation values for the VTAE, LNN, and NN are very similar, as shown in the image, as the three algorithms are closely clustered together on the graph, significantly diverging from the values obtained in the real pilot.

As shown in the accompanying table, none of the simulations achieved the task completion goal before 12:00; however, the VTAE simulation recorded the highest task completion ratio (TCR) among them. In contrast, the real-world Deusto experiment not only reached but exceeded the workload limitation. This indicates a higher-than-expected level of user engagement and motivation during the simulation. The real-world experiment concluded at 11:15, surpassing the task completion target, while the simulations had not met this goal by the end of the campaign at 12:00. The increased motivation of the participants contributed to a rise in variance in the real-world pilot. However, as previously noted, this variance is not unbounded; it represents a controlled increase that diminishes rapidly.

In conclusion, the experiment was significantly influenced by several factors, including the high motivation and engagement of users, who displayed a strong determination to complete the task within a short timeframe. The progression of tasks revealed an unexpectedly high level of user involvement and commitment, likely driven by the novelty effect or the ”Wow” effect. Despite this heightened participation, the variance observed in the real experiment remained within manageable levels. While certain aspects of the simulation align with the real experiment, an analysis of the metrics in Table 5 and Figure 7 indicates that the simulation’s behavior does not accurately replicate human behavior. This discrepancy arises from the inherent complexity of human behavior, which cannot be fully captured in a simulated environment. Consequently, the simulated behavior diverges from the actual behavior observed in the algorithm, but both have similarities in maintaining the variance under controlled values.

### 7.2. Qualitative Results

This section describes the qualitative evaluation collected through the survey realized at the end of the pilot. As mentioned above, 12 users participated and were recruited using a convenience sampling method among the university staff. The questionnaire consisted of 27 questions, and its goal was to evaluate the users’ acceptance and adoption of the system and their perception of it.

The survey starts by requesting the demographic information, as outlined in Table 6. A significant majority of participants are employees of the university (83.3%), males (75%), with a high level of technological abilities (91.7%), who fall within the 36–50 age range (75%). An advanced level of technological proficiency indicates that the participant engages in activities such as making purchases online and using spreadsheets, while an intermediate level reflects tasks like browsing websites and watching videos on platforms like YouTube. The convenience sampling method employed for recruitment focused on the university staff, which led to a relatively homogeneous participant pool, as revealed by the demographic information.

Following the collection of demographic information, the participants responded to several questions to assess their satisfaction and interest in the activity. The four questions related to this topic, their options, and the results are displayed in Table 7. The results of Questions 1, 2, and 3 show that the activity was enjoyable and valuable for the participants, as the means of the results are 4.42, 4.57, and 4 over 5, with 1 indicating the user strongly disagrees (unsatisfied) and 5 indicating the user strongly agrees. In the case of the open question (Question 4), some participants expressed confusion regarding the goal of the activity, and they encouraged us to improve this part of the presentation. Another recommendation proposed creating challenges for each task or gamifying the bot. These comments reflect that users are interested in the experiment but see possible improvements. This perspective is good; it proves that they see potential and possible applications.

The subsequent questions focused on system adoption and potential future use. These questions, their possible options to answer, and the answers are represented in Table 8. Based on Questions 1, 3, and 5, the future adaptation of the system by the participants is proven. With free text (Questions 2 and 4), users expressed what they need to ensure future adoption and their desire for improvement. Among these responses, some users expressed concerns about the bot’s battery consumption, while others suggested incorporating rewards or challenges for completing tasks or enabling the bot for additional purposes. These comments indicate a desire for further enhancements, reflecting the users’ interest in future improvements and use. The user adoption was positive, with many looking forward to more advanced features in the system.

The subsequent questions focused on the system’s customization and users’ perceptions. This set comprised five questions, whose answers are shown in Table 9. The scores slightly exceed the midpoint, suggesting that users perceive a degree of adaptation and customization within the system. These results indicate that the system’s customization features are progressing in the right direction and hold promise for further refinement and enhancement. The relatively low scores were anticipated, as the only factor used for personalization was the user’s position. Consequently, the outcome aligns with expectations. The final pair of questions in this section delved deeper into the user perception of personalization. Valuable feedback was gathered to indicate future improvements, such as the ability to specify a preferred area of interest and system customization options to request recommendations for nearby locations due to tiredness or time constraints. These ideas point towards more dynamic and user-centered customization features. In conclusion, users perceive some personalization, but in the future, they will want more customization and flexible interfaces that allow them to define their actual preferences.

The subsequent section focused on a series of questions about the Telegram bot’s performance. The aim was to understand the bot’s usability strengths and weaknesses in order to assess whether this interaction mechanism would be valid for further experiments. All of these questions were open questions that allowed users to express their opinions freely. These questions are shown in Table 10. Several users reported occasional instances of slow system performance and longer-than-expected map loading times. This issue was attributed to the computational constraints (latency throughput) of the device running the API of the VTAE service, a known issue that users had been alerted about. In terms of further improvements, suggestions included developing a better Telegram keyboard system for potential replies, which could streamline interactions and make the bot more user-friendly. Another suggestion was to indicate the user’s position within the interaction to enhance the user experience. Even though users pointed out certain areas for improvement, all of them concluded that the system worked properly.

The final set of questions was posed in the context of the recommendation of the VTAE engine, shown in Table 11. Users provided valuable feedback and identified several areas for improvement, which can guide the system’s future development. All of these questions were free text to allow users to express their opinions freely. Many users desired more personalized experiences, which could be achieved by explicitly defining their interests. They suggested that allowing users to specify areas of interest or directly communicate their preferences would enhance the system’s relevance and usability. Furthermore, the feedback highlighted the potential for an engagement strategy, possibly through feedback mechanisms or gamification, to increase user interaction and satisfaction. Overall, the user feedback underscores the importance of customization and engagement in enhancing the recommender system’s effectiveness.

The qualitative findings from the Deusto experiment offer valuable insights into user experience and potential system enhancements. The demographic data revealed a homogeneous participant pool, primarily comprising technically proficient individuals. The survey results indicated significant interest in the activity and adoption while also suggesting multiple opportunities for system improvement, particularly in the area of personalization. As the system currently lacks extensive customization based on user features, the perception of its customization was rated as average, as anticipated. However, users did not express negative comments regarding the task selection, indicating user comfort. Despite user comments and suggestions, the overall sentiment suggests user comfort and a desire for sustained system use, reflecting a positive outlook toward continued system utilization.

## 8. Conclusions of the Experiments

This section evaluates the results obtained from the simulation and Deusto experiments. It also evaluates their limitations and explains the qualitative and quantitative data of the experiments.

The quantitative evaluation of this work was grounded in both simulations and the quantitative analysis derived from the Deusto experiment. The results demonstrate that the VTAE maintains a low variance in task distribution, even as the number of tasks performed increases. Although the task completion rate (TCR) of the VTAE is occasionally surpassed by the nearest neighbor (NN) algorithm, the high variance associated with NN renders it unsuitable for this context. The exponential variance observed in the NN algorithm implies that data from one cell cannot be reliably compared with data from another, resulting in irrelevant final outcomes. Furthermore, the VTAE consistently exhibits a higher number of tasks performed over time, along with a lower variance and a higher TCR compared to the limited nearest neighbor (LNN) algorithm. Thus, from a quantitative perspective, the VTAE not only meets the stated objectives but also demonstrates efficiency in reallocating tasks within the context of altruistic participation.

Regarding the qualitative evaluation, conclusions are drawn from the questionnaires completed by participants in the Deusto experiment. While this experiment is limited by a small and demographically homogenous user group, it provides valuable insights into the behavior of the VTAE concerning personalization and user adoption. The evaluation offers a more in-depth understanding of user interaction with the system, allowing participants to observe the algorithm’s behavior and provide feedback on potential improvements. The qualitative findings from Deusto highlight a significant interest in the activity and the system’s adoption while also identifying opportunities for enhancement, particularly in terms of personalization. Although the system currently offers limited customization based on user attributes, the perceived level of personalization was rated as average, which aligns with expectations. Notably, users expressed no discomfort with the task recommendations, indicating overall satisfaction with the system. Despite some comments and suggestions for improvement, the general sentiment was positive, with users demonstrating comfort with the system and a willingness to continue using it. This suggests a promising outlook for sustained engagement.

Similarly, the simulation, in contrast to the real-world experiment conducted, reveals that, although simulations incorporate various variables related to human behavior, they differ significantly from what is observed in real-world scenarios. This is to be expected, as it is practically impossible to fully replicate the complexity of human behavior. Nevertheless, simulations are valuable for identifying and analyzing which human factors have the greatest impact on the performance of the VTAE, potentially limiting its ability to achieve its intended objectives. For instance, when comparing the results of Experiment 3 in the simulation (Section 6.3) with those of Experiment 2 (Section 6.2), it is evident that the user task completion probability variable has a greater impact on the TCR metric than the user availability probability variable. This insight suggests that it is preferable for users to avoid declaring their availability unless they are confident about completing the assigned tasks. Communicating this approach to users could help mitigate the negative impact of accepted but uncompleted tasks on the performance of the VTAE. Given that community participation is a critical factor in citizen science (CS) systems, this finding, derived from simulations, could indicate strategies to improve performance in real-world scenarios.

In summary, as demonstrated in the comparison in Section 7.1, the simulation did not fully capture the behavior observed in real-world scenarios. This is not surprising, as the complexity and variability of human behavior cannot be fully simulated. However, the insights gained from simulations are invaluable for understanding critical parameters and designing interventions to enhance the system’s effectiveness. While simulations cannot replicate the nuances of human behavior, they are instrumental in identifying key factors that influence the performance of systems like the VTAE. By leveraging these insights, it is possible to refine real-world applications, improve user participation strategies, and ultimately enhance the overall effectiveness of citizen science systems.

## 9. Conclusions and Future Work

Integrating users or non-expert citizens in the systems’ decision-making creates new challenges that create significant potential for new solutions. This work addresses the problem of spatial crowdsensing, more specifically concrete task allocation, in the context of altruist participation. Key challenges associated with altruist participation include the possible decrease in participation or the loss of the user’s interest due to work overload. In addition, the typical approach that promotes participation due to financial reward cannot be used in this context. This paper presents a new solution, the volunteer task allocation engine (VTAE), to address these challenges. This solution is not based on economic incentives, and it has two primary goals. The first one is to improve user experience by limiting the workload and creating a user-centric task allocation resolution. The second goal is to create an equal distribution of the tasks over the spatial distribution to make the solution robust against the possible decrease in participation.

This study underscores the significance of user-centered design in citizen science applications, particularly in contexts involving altruistic participation. The results demonstrate that the VTAE engine effectively ensures an equitable distribution of tasks across various locations, integrating participants into the decision-making process and reinforcing their role in task allocation solutions.

## Figures and Tables

**Table 1:** Glossary of the mathematical variables employed in the formulation and the VTAE.

Variable	Definition
m	Number of cells of a campaign.
n	Number of slots of a campaign.
L	Set of cells of a campaign.
$L_{j}$	The j-th cell of a campaign.
S	Set of slots of a campaign.
$S_{i}$	The i-th slot of a campaign.
$d_{s}$	Duration of the slot (except the last).
$M_{s}$	Maximum number of completed tasks allowed for each cell within each slot.
$St(S_{i})$	Starting time of the slot $S_{i}$.
$Ft(S_{i})$	Ending time of the slot $S_{i}$.
$P_{j}^{t}$	Priority of the cell $\left(L_{j}\right)$ at time t.
$t_{j,i}^{k}$	The k-th task of the cell $\left(L_{J}\right)$ at slot $S_{i}$.
$accep_{j}^{t}$	Number of accepted tasks for cell $\left(L_{j}\right)$ at the moment t.
$rea_{j}^{t}$	Number of realized task at cell $\left(L_{j}\right)$ at time t.
$C_{d}$	The distance between the centers of two adjacent cells.

**Table 2:** TCR evaluation over the different numbers of users (100, 75, 50, and 25) using the VTAE, LNN, and NN task allocation algorithms. The results are the mean of five executions. Numbers highlighted in bold indicate the highest TCR results by slot and number of users across the algorithms tested.

		Number of Users			
		**100 Users**	**75 Users**	**50 Users**	**25 Users**
Slot 1	VTAE	100%	100%	**94.52%**	**50.48%**
	LNN	100%	98.56%	74.95%	40.59%
	NN	**160.96%**	**121.22%**	83.40%	39.89%
Slot 2	VTAE	100%	100%	**95.43%**	**49.15%**
	LNN	100%	98.67%	73.46%	39.521%
	NN	**157.13%**	**122.02%**	82.29%	41.12%

**Table 3:** TCR evaluation over the different user availability probabilities using the VTAE, LNN, and NN task allocation resolution. Bold numbers indicate the highest TCR results by slot and available user rate for the tested algorithms.

		User Available Rate		
		**75%**	**50%**	**25%**
Slot 1	VTAE	100%	**94.52%**	**70.27%**
	LNN	91.7%	74.95%	48.62%
	NN	**101.9%**	83.40%	49.04%
Slot 1	VTAE	100%	**95.43%**	**61.38%**
	LNN	90.7%	73.46%	48.62%
	NN	**102.2%**	82.29%	46.33%

**Table 4:** TCR evaluation over the different task completion probabilities using the VTAE, LNN, and NN. Bold numbers indicate the highest TCR results for each slot and task completion rate among the tested algorithms.

		Task Completion Rate		
		**75%**	**50%**	**25%**
Slot 1	VTAE	**94.52%**	**66.22%**	**34.41%**
	LNN	74.95%	51.33%	25.05%
	NN	83.40%	49.10%	23.62%
Slot 2	VTAE	**95.43%**	**62.45%**	**31.33%**
	LNN	73.46%	52.45%	24.63%
	NN	82.29%	50.43%	26.22%

**Table 5:** TCR metric obtained in the Deusto experiment and in the simulations of this campaign using the VTAE, LNN, and NN algorithms. Additionally, two more metrics were added, namely the mean and the standard deviation of the variance metrics (Equation (8)) and the number of tasks completed per minute (Equation (10)). The numbers in bold represent the highest results for each metric among the tested algorithms.

		TCR	Mean Variance ± SD	Mean Task Completion ± SD
Real-world	Deusto Pilot	**111.90**%	1.03 ± 0.23	**0.97 ± 2.41**
Simulations	VTAE	73.81%	**0.42 ± 0.02**	0.65 ± 0.76
	LNN	70.24%	1.64 ± 0.72	0.62 ± 0.58
	NN	57.14%	1.64 ± 1.62	0.51 ± 0.47

**Table 6:** Demographic information of the Deusto experiment.

		%	N. of People
Age			
	19–35	25%	3
	36–50	75%	9
	0thers	0%	0
Gender			
	Female	25%	3
	Male	75%	9
	Other	0%	0
Level of education			
	PhD	58.3%	7
	Undergraduate	8.3%	1
	Master’s degree	33.3%	4
	Others	0 %	0
Technological knowledge			
	Intermediate Level	8.3%	1
	Advanced Level	91.7%	1
	Others	0	0
Work Status			
	Unemployed	8.3%	1
	Student	8.3%	1
	Employed	83.3%	10
	Others	0 %	0

**Table 7:** Inquiries about the participants’ interest level in the Deusto experiment. This table shows the users’ questions, options, and answers.

**Question 1.** I think this activity was interesting to me.							
Options:	Disagree	1	2	3	4	5	Agree
**Answers:**		0	0	1	5	6	
Mean ± SD: 4.42 ± 0.41							
**Question 2.** I think this activity was useful to me.							
Options:	Disagree	1	2	3	4	5	Agree
**Answers:**		0	1	6	2	3	
Mean ± SD: 3.58 ± 0.91							
**Question 3:** I think that this activity dealt with an important topic for me.							
Options:	Disagree	1	2	3	4	5	Agree
**Answers:**		0	1	3	3	5	
Mean ± SD: 4 ± 1							
**Question 4:** Comments or recommendations regarding this activity that you would like to share.							

**Table 8:** Inquiries concerning the system’s adoption by participants and their perception of it. This table shows the users’ questions, options, and answers.

**Question 1.** Do you see yourself continuing to use the recommender system and in the future?							
Options:	Yes	Maybe	No				
**Answers:**	5	5	2				
**Question 2.** If no, what would be the primary reason for discontinuing the use of the recommender system?							
**Question 3.** How likely are you to adopt the telegram bot or participate in the next pilot?							
Options:	Disagree	1	2	3	4	5	Agree
**Answers:**		1	0	1	2	8	
Mean ± SD: 4.33 ± 1.39							
**Question 4.** What factors influence your intention to adopt the bot services in your daily life?							
**Question 5.** Would you recommend the recommendation system to others? Or to use in other applications or propose?							
Options:	Yes	Maybe	No				
**Answers:**	7	5	0				

**Table 9:** Questions related to the users’ perceived customization of the system. This table shows the users’ questions, options, and answers.

**Question 1.** How personalized do you find the recommendations provided by the Telegram bot?							
Options:	Not at all	1	2	3	4	5	Extremely
**Answers:**		0	2	4	3	3	
Mean ± SD: 2.58 ± 1.07							
**Question 2.** To what extent do you feel that the recommendations align with your preferences?							
Options:	Not at all	1	2	3	4	5	Completely
**Answers:**		0	2	4	4	2	
Mean ± SD: 3.5 ± 0.92							
**Question 3.** How do you perceive the role of personalized recommendations in enhancing your overall experience with the Telegram bot?							
Options:	Little	1	2	3	4	5	Extremely
**Answers:**		1	0	5	5	1	
Mean ± SD: 3.42 ± 0.91							
**Question 4.** Are there any specific instances where you felt the recommendations were particularly well-tailored to your needs?							
**Question 5.** What improvements, if any, would you suggest to enhance the personalization of recommendations provided by the Telegram bot?							

**Table 10:** Feedback about the Telegram bot.

**Question 1.** What works well?
**Question 2.** What does not work?
**Question 3.** What could be improved/added?
**Question 4.** What is missing?
**Question 5.** How would you improve it?

**Table 11:** Recommendations and feedback information.

**Question 1.** Is there anything else you would like to share about your experience with the recommender system?
**Question 2.** What works well?
**Question 3.** What could be improved/added?
**Question 4.** What is missing?
**Question 5.** How would you improve it?

## Data Availability

The data can be found in the GIT repository https://github.com/mpuerta004/RecommenderSystem, accessed on 15 December 2024.
